# Nicotinic Acetylcholine Receptor Density in Cognitively Intact Subjects at an Early Stage of Parkinson’s Disease

**DOI:** 10.3389/fnagi.2014.00213

**Published:** 2014-08-14

**Authors:** Ioannis Ugo Isaias, Jörg Spiegel, Joachim Brumberg, Kelly P. Cosgrove, Giorgio Marotta, Naoya Oishi, Takahiro Higuchi, Sebastian Küsters, Markus Schiller, Ulrich Dillmann, Christopher H. van Dyck, Andreas Buck, Ken Herrmann, Susanne Schloegl, Jens Volkmann, Michael Lassmann, Klaus Fassbender, Reinhard Lorenz, Samuel Samnick

**Affiliations:** ^1^Department of Neurology, University of Würzburg, Würzburg, Germany; ^2^Department of Neurology, Saarland University, Homburg/Saar, Germany; ^3^Department of Nuclear Medicine, University of Würzburg, Würzburg, Germany; ^4^Department of Psychiatry, Yale University School of Medicine, New Haven, CT, USA; ^5^Department of Nuclear Medicine, Fondazione IRCCS Ca’ Granda Ospedale Maggiore Policlinico, Milan, Italy; ^6^Human Brain Research Center, Kyoto University Graduate School of Medicine, Kyoto, Japan

**Keywords:** 5IA-SPECT, nicotinic receptors, Parkinson disease, cognitive decline, dopamine acetylcholine

## Abstract

We investigated *in vivo* brain nicotinic acetylcholine receptor (nAChR) distribution in cognitively intact subjects with Parkinson’s disease (PD) at an early stage of the disease. Fourteen patients and 13 healthy subjects were imaged with single photon emission computed tomography and the radiotracer 5-[^123^I]iodo-3-[2(*S*)-2-azetidinylmethoxy]pyridine ([^123^I]5IA). Patients were selected according to several criteria, including short duration of motor signs (<7 years) and normal scores at an extensive neuropsychological evaluation. In PD patients, nAChR density was significantly higher in the putamen, the insular cortex and the supplementary motor area and lower in the caudate nucleus, the orbitofrontal cortex, and the middle temporal gyrus. Disease duration positively correlated with nAChR density in the putamen ipsilateral (ρ = 0.56, *p* < 0.05) but not contralateral (ρ = 0.49, *p* = 0.07) to the clinically most affected hemibody. We observed, for the first time *in vivo*, higher nAChR density in brain regions of the *motor* and *limbic* basal ganglia circuits of subjects with PD. Our findings support the notion of an up-regulated cholinergic activity at the striatal and possibly cortical level in cognitively intact PD patients at an early stage of disease.

## Introduction

At an early motor stage, Parkinson’s disease (PD) is predominantly characterized by a progressive loss of the nigrostriatal dopaminergic neurons leading to a severe state of dopamine depletion. In addition to the decline in dopaminergic function, other neurotransmitter systems are involved in PD, including the nicotinic cholinergic system (Jellinger, 1991; Posadas et al., 2013). Indeed, anti-cholinergics were the first widely accepted treatment for parkinsonism. In 1867, Ordenstein first reported their antiparkinsonian effect, which Charcot had discovered fortuitously when administering tinctures of deadly nightshade (*Atropa belladonna*) for excessive salivation in parkinsonian patients (Lang and Blair, 1989).

The two primary sources of acetylcholine (ACh) in the brain include local interneurons that are interspersed among their cellular targets and projection neurons that innervate distal areas. Most brain regions, including the pedunculopontine and laterodorsal tegmental areas, belong to the latter category, whereas the former includes the striatum and nucleus accumbens. ACh signals through two classes of receptors localized both pre- and postsynaptically: metabotropic muscarinic acetylcholine receptors (mAChRs) and ionotropic nicotinergic acetylcholine receptors (nAChRs). Presynaptic mAChRs (M2, M4) act as inhibitory autoreceptors on the cholinergic terminals, with M4 predominant in striatum. Postsynaptic mAChRs can be either inhibitory (M2, M4) or excitatory (M1, M3, M5). Although the actual mechanism of action in PD is not known, clinically available anti-cholinergics (e.g., trihexyphenidyl, benztropine, etc.) act mainly as competitive antagonists of mAChRs (Brocks, 1999). The nAChRs are pentameric ligand-gated ion channels composed of α-subunits (homomeric receptors) or of α- (α2–α7) and β-subunits (β2–β4) (heteromeric receptors) (reviewed in Gotti and Clementi, 2004). Presynaptic nAChRs induce release of a number of neurotransmitters, including dopamine. Postsynaptic nAChRs depolarize neurons, increase their firing rate, and can contribute to long-term potentiation (reviewed in Picciotto et al., 2012).

The striatum is a nodal structure of the basal ganglia circuits and one of the brain areas with the highest concentration of markers of cholinergic transmission. Large aspiny cholinergic interneurons (ChIs) constitute <2% of the entire striatal neuronal population but exert a powerful influence on its output, which is mediated by the medium spiny neurons (MSNs). Dopamine depletion elicits an increased excitability of ChIs, mainly due to the removal of D2-mediated inhibitory control (Maurice et al., 2004). In addition, rhythmic firing of the ChIs and breakdown of autoinhibition of ACh release by M4 results in the unregulated release of ACh, which selectively increases excitability of MSNs, particularly those of the indirect pathway (Aosaki et al., 1995; Kreitzer and Malenka, 2007). Increased cholinergic activity in subjects with PD was not confirmed by anatomopathological studies that documented mainly an extensive nAChR reduction in the brain of patients with PD (Perry et al., 1989; Rinne et al., 1991; Aubert et al., 1992). *In vivo* studies in non-demented PD subjects are instead limited and controversial. Molecular imaging using either 2-[^18^F]FA-85380 and positron emission tomography (PET) or [^123^I]5IA and single photon emission computed tomography (SPECT) demonstrated variably reduced nAChRs density in cortical areas only (i.e., frontal and parietal lobes), amygdala (Fujita et al., 2006; Oishi et al., 2007; Meyer et al., 2009) or in the striatum and substantia nigra (Kas et al., 2009).

In the present study, we investigated by means of 5-[^123^I]iodo-3-[2(*S*)-2-azetidinylmethoxy]pyridine ([^123^I]5IA), a specific α4β2 nAChR ligand, and SPECT ([^123^I]5IA-SPECT) a group of PD patients specifically selected for a short disease duration, the capability to be withdrawn from all dopaminergic medications for 3 days, and normal scores from extensive neuropsychological evaluation.

## Materials and Methods

Healthy subjects were enrolled at the Yale University School of Medicine with the approval of the Yale Human Investigation Committee, the West Haven Veterans Administration Human Subjects Subcommittee, the Radiation Safety Committee, and the Food and Drug Administration. [^123^I]5IA-SPECT in patients with PD was performed at the University Hospital of Würzburg and approved by the University Hospital of Würzburg and the German Federal Office for Radiation Protection (Bundesamt für Strahlenschutz, Salzgitter, Germany). All participants gave written informed consent.

### Subjects

The study involved 14 subjects with PD (8 males; median age: 64 years, range: 52–78 years; recruited at Saarland University) and a control group of 13 neurologically intact adults (5 males; median age, 61 years, range: 51–78 years; recruited at Yale University). Median age of PD patients at motor symptoms onset was 60 years (43–75 years). The diagnosis of PD was made according to the UK Parkinson Disease Brain Bank criteria and patients evaluated with the Unified Parkinson Disease Rating Scale (UPDRS). Median UPDRS-III (motor part) score was 21 (range, 9–33) in meds-off phase (12 h l-DOPA withdrawal; selegiline, rasagiline, amantadine, cabergoline, pergolide, and prolonged formulations of dopamine agonists were discontinued for 72 h). The right hemibody showed more severe akinetic–rigid signs in all but two PD patients (UPDRS-22 median score right: 3 and left: 1; UPDRS-23/24/25 grand median score right: 1 and left 0). The average l-DOPA daily dose was 114 ± 147 mg and the average l-DOPA equivalent daily dose (LEDD) was 626 ± 412 mg. All patients had a positive response to dopaminergic drugs. Clinical inclusion criteria for subjects with PD were as follows: (1) UPDRS part I score of 0; (2) disease duration <7 years (anamnestic to first motor symptoms onset); (3) Hoehn and Yahr scale, stage 2; (4) no psychiatric disorders or other neurological diseases other than PD; and (5) absence of any signs indicative for atypical parkinsonism (e.g., gaze abnormalities, autonomic dysfunction, psychiatric disturbances, etc.). All subjects had no cognitive decline as well as no deficit in visual attention, task switching, memory, or learning strategies, as assessed by the Mini Mental State Examination (score >27), Word list (Learning recall, Intrusions, Savings, and Recognition), Visuoconstructive Ability (Figure drawing, Figure recall, and Figure savings), Verbal Fluency Test, Modified Boston Naming Test, Phonemic Fluency, and Trail-making test A and B. Patients were excluded from the study if they were taking cholinergic or anti-cholinergic drugs. Further exclusion criteria were pregnancy or breastfeeding, a partner who was capable of childbearing and smoking in the last 5 years.

None of the controls had a history of neurological disorders, head trauma with loss of consciousness, epilepsy, brain surgery, or excessive drug or alcohol consumption at any time during their life. All participants gave written informed consent following a protocol approved by the local institutional Ethics Committee.

### Radiochemistry

[^123^I]5IA was prepared by [^123^I]iododestannylation of the corresponding stannyl precursor 5-(tri-*n*-butylstannyl)-3-([1-*t*-butoxycarbonyl-2(*S*)-azetidinyl]pyridine as described previously (Lorenz et al., 2014). In details, to a 1.5-ml conical vial (Pierce, Sankt Augustin, Germany) containing carrier-free sodium [^123^I]iodide (typically 300–500 MBq in 50–150 μl 0.02 N NaOH; GE Healthcare, Braunschweig, Germany) were added in the following order: 50 μg of 5-SnBu3-A85380 dissolved in 25 μl of ethanol, 5 μl 1 M HCl, and 15 μg chloramine-T (in 10 μl H2O). After 30 min incubation at 40°C, 30 μl of trifluoroacetic acid was added to the mixture and the sealed vial heated for additional 15 min at 100°C. After cooling at room temperature, the mixture was made alkaline by adding 150 μl of 1 M aqueous K2CO3. [^123^I]5IA was then isolated from the starting materials and radioactive impurities by reverse-phase high-performance liquid chromatography (HPLC), using a reversed phase RP-C18 column (Nucleosil 100-7, 250 mm × 4 mm; CS-Chromatography Service, Germany) and water/ethanol/H3PO4 (90:10:0.1, v/v/v), as eluent at a flow rate of 1.3 ml/min, while monitoring ultraviolet (UV) at 254 nm with a UV detector (HP1100) and radioactivity with a scintillation detector (Berthold, Wildbad, Germany), respectively. The fraction containing [^123^I]5IA was collected into a sterile tube, buffered with PBS (pH 7.0) under sterile conditions and sterile-filtered through a 0.22-μm filter (Millipore, Eschborn, Germany) into an evacuated sterile vial for investigation. [^123^I]5IA was obtained with an isolated radiochemical yield of 85 ± 6% (*n* = 30) and a radiochemical purity >99%. The specific activity as determined from the UV absorbance at 254 nm exceeded 125 GBq/μmol (the detection limit of our system). The injection solution was additionally quality controlled by HPLC [Nucleosil RP-100-7, 250 mm × 4 mm; water/ethanol/phosphoric acid, 90:10:0.1 (v/v/v) at 1.3 ml/min].

### Image data acquisition and analysis

Patients fasted at least 4 h before [^123^I]5IA administration. Possible iodine uptake by the thyroid was blocked by oral medication with sodium perchlorate (Irenat^®^, Bayer, Leverkusen, Germany). Patients received 185 ± 5 MBq of freshly prepared [^123^I]5IA intravenously over 60 min. The above described radiosynthesis provided [^123^I]5IA in form of carrier-free (n.c.a.) tracer, with the highest possible specific activity. The approximate administrated mass in an injectable solution with 185 MBq of [^123^I]5IA was <0.001 nmol (<1 pmol).

Cerebral SPECT imaging was acquired with a dual-head gamma camera (E.Cam Duet, Siemens Medical Solutions, Hoffman Estates, IL, USA) equipped with medium energy collimators. At 2 and 4 h after injection of [^123^I]5IA, 120 views (40 s per view) were acquired over a 360° circular orbit, and reconstructed into a 128 × 128 matrix with a pixel size of 3.9 mm and slice thickness of 3.9 mm. Imaging at 4 h after injection was chosen in accordance with previous kinetic modeling data in healthy volunteers (Oishi et al., 2007; Cosgrove et al., 2012) and for practical reasons concerning scanning the patients. Reconstruction was performed with filtered back-projection with a Butterworth filter (order 8, cutoff 0.4) followed by attenuation correction according to the Chang method (Chang, 1978), with an attenuation coefficient of 0.11/cm to generate the transversal slices.

For further data analysis, the reconstructed transverse sections were transferred to a Hermes workstation (Hermes Medical Solutions, Stockholm, Sweden). Brain regions were analyzed using the brain analysis program BRASS (version 3.5, Hermes Medical Solutions, Stockholm, Sweden). Each image volume was recorded to match to the built-in ECD template using an affine transform (nine parameters). Manual fitting was necessary, because of the low background uptake resulting in insufficient contrast for automatic delineation of the brain contour. The ECD template consisted of a three dimensional region of interest (ROI) map of 46 predefined brain regions. The mean count per voxel was determined for each region in both hemispheres. The normalized data were calculated as the ratio of mean count per voxel to mean count per voxel of the global [^123^I]5IA brain uptake (= average of all 46 measured brain regions) for each region and each subject. We selected as a reference the whole brain uptake as the most conservative approach (Terrière et al., 2010). We also performed a statistical parametric mapping (SPM version 8, Wellcome Department of Cognitive Neurology, UCL, London, UK) analysis. This method allows exploratory voxel-by-voxel group comparisons throughout the entire brain volume without requiring an *a priori* hypothesis. The template for SPM analysis was provided by Oishi et al. (2007). Of relevance to this manuscript, we performed a voxel-based analysis applying the proportional scaling global mean, thresholded at *p* < 0.05 and corrected for Family wise-error (FWE). Brain regions (approximate Brodmann areas) were estimated based on the methods of Talairach and Tournoux (1988) after adjustment (www.mrc-cbu.cam.ac.uk/Imaging/mnispace.html) for differences between MNI and Talairach coordinates.

### Statistical analysis

Normality of data distribution was tested by the Shapiro–Wilks test. Gender distribution among groups was tested with chi-square. Demographic and brain imaging data were compared by means of Wilcoxon rank-sum test. Spearman’s Rho test was used to identify correlations among [^123^I]5IA binding values and clinical and demographic data. Statistical analyses were performed with the JMP statistical package, version 8.0.2 (SAS Institute).

## Results

**Figure 1:**
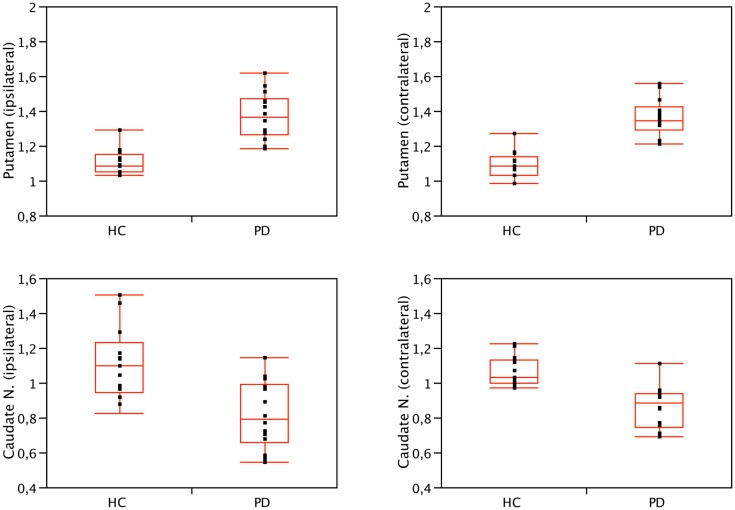
**Binding values of nAChRs of the caudate nucleus and putamen of PD patients and HC**. Compared to controls, nAChRs density was bilaterally lower in the caudate nucleus (*p* < 0.01, Wilcoxon rank-sum test) and higher in the putamen of PD patients (*p* < 0.001, Wilcoxon rank-sum test). No significant difference was found when comparing ipsilateral and contralateral (or left and right) side, both in patients and controls. Contralateral refers to the side opposite to the clinically most affected hemibody. Right is conventionally contralateral for HC.

We found significant differences in [^123^I]5IA binding values within the striatum of cognitively intact PD patients at an early disease stage compared to controls. In particular, the putamen showed a higher density of nAChRs bilaterally (1.36 ± 0.1 vs. 1.08 ± 0.1, contralateral to the clinically most affected hemibody and 1.37 ± 0.13 vs. 1.09 ± 0.09, ipsilateral; *p* < 0.001 all), whereas the caudate nucleus had a lower nAChRs density (0.86 ± 0.12 vs. 1.04 ± 0.15, contralateral and 0.81 ± 0.2 vs. 1.11 ± 0.21 ipsilateral; *p* < 0.01 all) (Figure 1). Two functionally correlated cortical areas, namely the insular cortex and the orbitofrontal cortex showed higher and lower nAChR density, respectively (Table 1). Disease duration positively correlated with nAChR density in ipsilateral (ρ = 0.56, *p* < 0.05) but not contralateral putamen (ρ = 0.49, *p* = 0.07) (Mitsis et al., 2009a). This correlation was statistically significant also when weighted for age and disease duration.

**Table 1:** **Binding values of nAChRs**.

	PD	HC	*p* Value
Putamen C	1.36 ± 0.10	1.08 ± 0.10	<0.0001
Putamen I	1.37 ± 0.13	1.09 ± 0.09	<0.0001
Caudate C	0.86 ± 0.12	1.04 ± 0.15	<0.01
Caudate I	0.81 ± 0.20	1.11 ± 0.21	<0.01
Thalamus C	1.68 ± 0.23	1.67 ± 0.18	0.46
Thalamus I	1.62 ± 0.24	1.68 ± 0.20	1.00
Sensorimotor L	1.07 ± 0.07	1.05 ± 0.09	0.40
Sensorimotor R	1.02 ± 0.07	1.02 ± 0.08	0.88
Frontal lobe L	0.93 ± 0.06	0.95 ± 0.08	0.19
Frontal lobe R	0.92 ± 0.06	0.95 ± 0.09	0.11
Orbitofrontal L	0.85 ± 0.08	1.00 ± 0.11	<0.001
Orbitofrontal R	0.83 ± 0.10	1.00 ± 0.10	<0.001
Temporal lobe L	0.97 ± 0.05	0.96 ± 0.08	0.73
Temporal lobe R	0.94 ± 0.05	0.96 ± 0.08	0.05
Parieto-temporal L	1.00 ± 0.04	0.90 ± 0.07	0.16
Parieto-temporal R	0.94 ± 0.04	0.96 ± 0.08	0.43
Insular cortex L	1.13 ± 0.09	0.95 ± 0.09	<0.001
Insular cortex R	1.13 ± 0.10	0.91 ± 0.07	<0.001
Gyrus cinguli L	0.85 ± 0.07	0.88 ± 0.10	0.16
Gyrus cinguli R	0.86 ± 0.09	0.90 ± 0.10	0.40
Occipital L	0.89 ± 0.05	0.86 ± 0.08	0.05
Occipital R	0.87 ± 0.05	0.85 ± 0.07	0.38
Cerebellum cortex L	1.01 ± 0.06	0.98 ± 0.01	0.22
Cerebellum cortex R	1.01 ± 0.06	0.98 ± 0.01	0.30
Cerebellum white matter L	1.12 ± 0.07	1.07 ± 0.04	0.17
Cerebellum white matter R	1.16 ± 0.07	1.10 ± 0.04	0.05
Pons and midbrain	1.33 ± 0.13	1.34 ± 0.09	0.69

*For the striatum and thalamus only we listed the binding values of contralateral (C) and ipsilateral (I) with respect to the clinically most affected hemibody. For healthy controls, left hemibody refers conventionally to ipsilateral. Other brain regions are listed as left (L) and right (R). *p* Value refers to Wilcoxon rank-sum test*.

**Figure 2:**
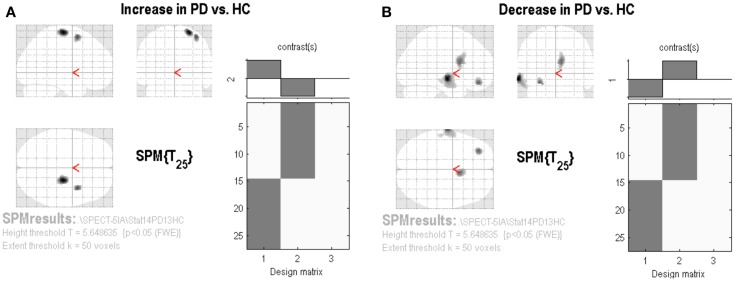
**Statistical parametric mapping results**. Group contrast giving relative increase **(A)** or decrease **(B)** in [^123^I]5IA uptake in PD patients applying the proportional scaling global mean, thresholded at *p* < 0.05 and corrected for FWE.

The SPM analysis confirmed a reduced nAChRs density in the (right) caudate nucleus (Table 2; Figure 2B). This analysis also showed in PD patients a lower density of nAChRs in left middle frontal gyrus (BA11) and left middle temporal gyrus (BA21) (Table 2; Figure 2B). On the contrary, cholinergic activity was increased in the supplementary motor area (SMA) (i.e., right precentral gyrus and right middle frontal gyrus, BA6) (Table 2; Figure 2A).

**Table 2:** **Brain regions of significantly correlation between the voxel-by-voxel [^123^I]5IA distribution in the PD group as compared with the control group in statistical parametric mapping (SPM8) analyses**.

Region – Brodmann area	Coordinate (Talairach)	*Z* score
**INCREASED**		
R precentral gyrus – BA6	24, −18, 68	5.22
R middle frontal gyrus – BA6	32, −1, 57	5.12
**DECREASED**		
R caudate nucleus	4, 7, 12	4.82
L middle frontal gyrus – BA11	−28, 41, −9	5.41
L middle temporal gyrus – BA21	−64, −12, −7	6.00
	−56, −2, −15	4.84
	−53, −22, −13	5.97

## Discussion

Despite the long history of the dopamine-ACh balance hypothesis, we are just beginning to understand why and how dopamine depletion triggers a profound deterioration of basal ganglia circuit dynamics, such as over-activation of cholinergic system activity leading to motor and cognitive disturbances.

Our findings support the notion of an up-regulated cholinergic activity at a striatal and possibly cortical level in cognitively intact PD patients at an early stage of disease. Higher nAChR density may occur as a compensatory mechanism to maintain dopaminergic tone, in particular in the putamen, the region with the most extensive loss of dopaminergic innervation at the early motor stage of disease (Isaias et al., 2007). Enhanced [^123^I]5IA binding was also present in the limbic cortex, a brain area possibly involved in the pathophysiology of PD at a pre-motor stage (Bohnen et al., 2010). The SPM analysis specifically showed an increased cholinergic activity in the SMA, which is considered a key structure of the cortico-basal ganglia motor loop (Nachev et al., 2008). This finding is of particular relevance given the functional connectivity between SMA and the putamen (Yu et al., 2013). Indeed, deep brain stimulation of the subthalamic nucleus, which is now an established surgical therapy in PD (Bronstein et al., 2011; Merola et al., 2011), as well as dopaminergic drugs, reduce blood flow (Hershey et al., 2003; Bradberry et al., 2012) and glucose metabolism (Trošt et al., 2006) in the SMA of PD patients. Of relevance, such an increased [^123^I]5IA binding was selectively contralateral to the less affected hemibody of PD patients. Thus further suggesting a putative cholinergic compensatory activity at a cortical level upon striatal dopaminergic innervation loss. Additional imaging studies, assessing both cholinergic and dopaminergic innervation changes, in the same PD patient, might further clarify dopamine-ACh interplay at different clinical stages. Of relevance, it is worth mentioning that [^123^I]5IA binding is an indirect measurement of cholinergic activity, as it describes nAChRs density mainly at a post-synaptic level. Other compounds, targeting acetylcholinesterase or vesicular acetylcholine transporters, might better elucidate an up-regulation of the cholinergic activity, especially at a pre-synaptic level (see later).

The caudate nucleus and the orbitofrontal cortex showed instead lower [^123^I]5IA binding. This finding may suggest a different dopamine-ACh balance in striatal areas that are less-deprived of dopamine (Isaias et al., 2007). However, it might also suggest a greater impairment of cholinergic innervation in the *orbitofrontal* basal ganglia circuit than in the *motor* or *limbic* circuits at an early stage of PD, even in cognitively intact PD patients. An association between anti-cholinergic drug use and cognitive decline in PD has been documented (Ehrt et al., 2010). It should be noted, however, that clinically available anti-cholinergics (e.g., trihexyphenidyl, benztropine, etc.) mainly act as competitive antagonists at mAChRs. A stepwise executive dysfunction has been described in cognitively intact PD patients (Taylor et al., 1986) who suffer damage to the frontal lobes and/or fibers connecting the frontal lobes with the head of the caudate during electrode implantation for deep brain stimulation (Okun et al., 2012). The role of [^123^I]5IA-SPECT as a screening tool for identifying patients at risk for (surgery-related) cognitive decline should be further investigated.

No previous studies have described higher nAChR binding leveling in PD patients compared to controls. Discrepancies might be related to the relatively short disease duration and the early disease stage of patients enrolled in this study. In addition, we carefully excluded patients with cognitive problems by means of an extensive neuropsychological evaluation (Mitsis et al., 2009b). We also enrolled patients able to stop their dopaminergic therapy for 3 days to limit a possible acute effect of dopaminergic drugs on nAChRs binding measurement. Indeed, l-DOPA treatment significantly decreased *in vitro* [^123^I]5IA binding in the striatum, but not in cerebral cortex in normal squirrel monkeys (Quik et al., 2003). Similarly, a high daily dose of dopamine agonist showed a significant negative correlation with density of nAChRs in the cerebellum, temporal, parietal, and occipital cortices (Oishi et al., 2007). In previous studies, dopaminergic drugs were not suspended (Fujita et al., 2006; Meyer et al., 2009) or stopped for 12 h only (Kas et al., 2009), despite the long half-life of some dopaminergic drugs (e.g., ergot derivatives) (Oishi et al., 2007). Still, even a drug-withdrawal of 72 h, as in our study, might not be sufficient in avoiding an acute drug effect on [^123^I]5IA binding, especially when patients are taking long-lasting dopaminergic drugs (e.g., dopamine agonists). A study on *drug naïve* patients is warranted, also to avoid a putative chronic effect of dopaminergic drugs on the striatal cholinergic system.

The majority of anatomopathological studies (Perry et al., 1989; Rinne et al., 1991; Aubert et al., 1992), but not all (Lange et al., 1993), reported a loss of nAChR agonist binding in the striatum of PD patients. Post-mortem findings are however not directly comparable with our results as they cannot detach possible compensatory changes early at a disease stage. In many cases, it is also unclear whether these studies have included demented patients (Rinne et al., 1991; Court et al., 2000).

Few PET studies with [^11^C]methyl-4-piperidinyl propionate acetylcholinesterase ([^11^C]PMP) (Gilman et al., 2010) but not others (Shinotoh et al., 1999; Bohnen et al., 2006, 2010) described a reduced striatal cholinergic activity in patients with PD. Indeed, in a large cohort of non-demented PD patients, cholinergic projection alterations, investigated by means of [^11^C]PMP and PET, were highly heterogeneous with over 65 out of 101 subjects with PD showing neocortical and thalamic acetylcholinesterase activity within the normal range (Bohnen et al., 2012). It is worth mentioning that [^11^C]PMP and PET does not allow accurate measurements of brain areas with high acetylcholinesterase activity levels, such as the striatum.

Last but not least, the uptake of [^123^I]iodobenzovesamicol (IBVM), an *in vivo* marker of the vesicular ACh transporter binding, was reduced only in parietal and occipital cortex (Kuhl et al., 1996) but not in the basal ganglia of non-demented PD patients.

Such a great variability in cholinergic activity in PD patients deserves further studies as it might unmask endogenous neuroprotective or compensatory mechanisms (Quik et al., 2012, 2013) and overall help profiling the disease changes at an early stage of the disease. In particular, there is increasing evidence that nicotine and other drugs that act at nAChRs may be beneficial in the management of PD. Several studies in animals have shown that nicotine administration enhances dopaminergic integrity in the striatum, especially when administered before/during but not after nigrostriatal damage (Huang et al., 2011). Indeed, a cholinergic loss does not parallel dopaminergic state in PD patients as measured by means of ^18^F-DOPA and PET (Kas et al., 2009) or with markers of disease severity (i.e., UPDRS-III), disease duration, and daily dose of l-DOPA and dopamine agonists (i.e., LEDDs) (our study, Bohnen et al., 2006; Oishi et al., 2007; Kas et al., 2009).

Finally, several limitations of this study must be acknowledged. In particular: (1) there is no region devoid of nAChRs and therefore we could not measure accurately non-specific binding and then calculate a specific binding for the striatal area; (2) the low resolution of SPECT and the different methodological techniques in acquiring brain images might have contributed to the lack of correlations with markers of disease severity and progression or even account for discrepancies with previous studies; (3) ROIs were not drawn on single patient anatomical template (e.g., MRI-based); and (4) the several clinical inclusion criteria defined highly homogeneous cohorts but greatly limited the number of participants to this study. Despite these limitations, mostly related to the difficulty of applying a new radioactive compound for *in vivo* imaging studies, our findings provide relevant information of nAChR distribution at an early motor stage of PD.

## Author Contributions

Ioannis Ugo Isaias, Jörg Spiegel, Joachim Brumberg, Kelly P. Cosgrove, Markus Schiller, Ulrich Dillmann, Christopher H. van Dyck, Andreas Buck, Ken Herrmann, Susanne Schloegl, Jens Volkmann, Michael Lassmann, Klaus Fassbender, Reinhard Lorenz, and Samuel Samnick designed, analyzed, and performed research. Giorgio Marotta, Naoya Oishi, Takahiro Higuchi, and Sebastian Küsters performed data analysis. All authors contributed to and have approved the final manuscript.

## Conflict of Interest Statement

The authors declare that the research was conducted in the absence of any commercial or financial relationships that could be construed as a potential conflict of interest.
